# Antiviral Activity of Ailanthone from *Ailanthus altissima* on the Rice Stripe Virus

**DOI:** 10.3390/v16010073

**Published:** 2023-12-31

**Authors:** Qingwei Tan, Jianxuan Zhu, Yuanyuan Ju, Xinlin Chi, Tangdan Cao, Luping Zheng, Qijian Chen

**Affiliations:** 1 Key Laboratory of Biopesticide and Chemical Biology, Ministry of Education, Fujian Agriculture and Forestry University, Fuzhou 350002, China; 17793649297@163.com (J.Z.); sunshinejyy520@163.com (Y.J.); chixinlin@foxmail.com (X.C.); d18216469856@163.com (T.C.); lupingz@126.com (L.Z.); 2 Institute of Plant Virus Research, Fujian Agriculture and Forestry University, Fuzhou 350002, China

**Keywords:** rice stripe virus, ailanthone, quassinoid, coat protein, movement protein, in vivo antiviral

## Abstract

Rice stripe disease caused by the rice stripe virus (RSV), which infects many Poaceae species in nature, is one of the most devastating plant viruses in rice that causes enormous losses in production. Ailanthone is one of the typical C_20_ quassinoids synthesized by the secondary metabolism of *Ailanthus altissima*, which has been proven to be a biologically active natural product with promising prospects and great potential for use as a lead structure for pesticide development. Based on the achievement of the systemic infection and replication of RSV in *Nicotiana benthamiana* plants and rice protoplasts, the antiviral properties of Ailanthone were investigated by determining its effects on viral-coding RNA gene expression using reverse transcription polymerase chain reaction, and Western blot analysis. Ailanthone exhibited a dose-dependent inhibitory effect on RSV *NSvc3* expression in the assay in both virus-infected tobacco plants and rice protoplasts. Further efforts revealed a potent inhibitory effect of Ailanthone on the expression of seven RSV protein-encoding genes, among which *NS3*, *NSvc3*, *NS4*, and *NSvc4* are the most affected genes. These facts promoted an extended and greater depth of understanding of the antiviral nature of Ailanthone against plant viruses, in addition to the limited knowledge of its anti-tobacco mosaic virus properties. Moreover, the leaf disc method introduced and developed in the study for the detection of the antiviral activity of Ailanthone facilitates an available and convenient screening method for anti-RSV natural products or synthetic chemicals.

## 1. Introduction

Rice stripe virus (RSV), which causes rice stripe disease and results in enormous losses in production in eastern Asia, is a type of species in the genus Tenuivirus and is considered one of the most economically important virus pathogens of rice [1]. RSV infects many Poaceae species in nature, including several important crops such as rice (*Oryza* spp.), wheat (*Triticum aestivum* L.), barley (*Hordeum vulgare* L.), oat (*Avena sativa* L.), foxtail millet (*Setaria italica* L.), and sorghum (*Sorghum bicolor* L.) [2]. Moreover, RSV infects dicotyledonous *Arabidopsis thaliana* and *Nicotiana benthamiana*, which are widely used in the laboratory as model plants for virology studies [3,4,5]. In *N. benthamiana*, RSV can be transmitted from plant to plant by rub inoculation [6,7].

Effective management of plant virus disease has always been a problem and challenge in plant protection [8]. Due to the lack of highly effective antiviral agents, the use of insecticides against the small brown planthopper (SBPH), which is the main insect vector that transmits RSV, and the exploitation of genetically resistant cultivars are considered the most effective approaches at present to manage rice stripe disease [9,10]. Considering the great threat of plant virus diseases on crop production and the current deficiency in efficient control strategies, there is an urgent and sustained need to develop rapid, convenient, and effective models for screening antiviral agrochemical candidates. During the screening of antiviral natural products using the tobacco mosaic virus as the target pathogen, we reported the identification of quassinoids as major active antiviral structures from *Ailanthus altissima* [11]. In this study, we developed and conducted a series of antiviral assays based on the mechanical rubbing inoculation of RSV in *N. benthamiana* and polyethylene glycol-induced virus infection in rice protoplasts. We thus report herein the antiviral properties of Ailanthone, which is one of the typical quassinoids isolated from the MeOH extract of *A. altissima*. 

## 2. Materials and Methods

### 2.1. Sources of Virus, Vectors, and Plant Materials

Rice stripe virus-infected rice plants were collected from Jiangsu Province in China. The virus was confirmed by using a Western blot assay and maintained by successive transovarial infections in the small brown planthopper (SBPH) on rice seedlings in an insect-rearing room at 25 °C and 16 h light and 8 h dark conditions, at the Institute of Plant Virus Research, Fujian Agriculture and Forestry University.

The virus particle was isolated and purified from the newly emerged leaves of RSV-infected rice that had obvious and typical symptoms such as chlorosis, stunting, necrosis, or other symptoms using a procedure described by Wang et al. [12]. The purified virus was characterized by 12% SDS-polyacrylamide gel electrophoresis, and its concentration was determined using the Bradford assay with bovine serum albumin as a standard. Purified RSV was stored at −80 °C and always diluted to the same concentration before use to maintain the equivalent inoculation quantity.

RSV infection was achieved through the mechanical rubbing of *N. benthamiana* plants, according to the method employed by Xu and Zhou [7]. In brief, *N. benthamiana* with 6−8 fully expanded true leaves were used for experiments, and three of the top fully expanded leaves were used for virus infection. RSV infection was achieved by rubbing *N. benthamiana* leaves with crude extracts from RSV-infected *O. sativa* leaves ground in 0.2 M phosphate buffer.

The candidate compound used in the study, Ailanthone, was previously purified from MeOH extracts of *Ailanthus altissima* in our laboratory. 

### 2.2. Preparation of Rice Protoplasts and Virus Infection

Rice suspension cells were derived from germinal vesicles of *Oryza sativa* ssp. *Japonica* cv. *Nipponbare*, which was grown in N6 medium (pH 5.8) containing 2 mg/L 2,4-D and 0.4 g/L proline at 25 °C under rotation at 120 rpm. Protoplast isolation and virus inoculation were performed as described [13,14,15] with some modifications. The suspension cell was first digested with an enzyme mixture, and the undigested suspension cells were removed by a double filtration procedure with 100 µm and 40 µm nylon filters. The protoplasts were suspended in a washing solution (0.4 M mannitol, 6.8 mM CaCl_2_, pH 5.6), followed by centrifugation for 5 min at 100× *g*. The suspension–centrifugation cycle was repeated three times. The pelleted protoplasts were finally resuspended in protoplast suspension medium (0.4 M mannitol, 20 M CaCl_2_ 2H_2_O, 5 mM Mes-KOH, pH 5.7) and counted in a hemocytometer to prepare a density of 3~4 × l0^6^ protoplasts per ml. Then, 200 µL of protoplast solution was mixed with 1 µg RSV, and 220 µL of polyethylene glycol-CHS solution (0.4 M mannitol, 0.1 M CaCl_2_ 2H_2_O, 40% polyethylene glycol 4000) was added to the protoplasts and incubated for 20 min at room temperature. Then, the sample was washed with 1 mL of W5 buffer three times. The virus-infected protoplasts were resuspended in 1 mL of WI buffer and incubated in the dark at 28 °C.

### 2.3. RNA Isolation, cDNA Synthesis, and Quantitative Real-Time RT-PCR Analysis

Total RNA was extracted using the Plant RNA kit (Omega Bio-Tek, Norcross, GA, USA). The optical density of the RNA extracts was determined using an ultraviolet spectrophotometer, and total RNA purity was estimated by calculating OD_260_/OD_280_, which remained within 1.8 and 2.2. The concentration of total RNA was calculated according to the dilution ratio and the value of OD_260_.

cDNAs were synthesized using a StarScript II First-Strand cDNA Synthesis Kit with gDNA Remover (GenStar, Beijing, China). PCR amplifications were carried out using a 2× Taq PCR StarMix with Loading Dye (GenStar) and run according to the program as described (a. 2 min at 94 °C; b. 30 s at 94 °C; c. 30 s at 55 °C; d. 1 min at 72 °C; e. Repeat steps b–d for optimized cycles ranging from 12 to 28; f. 8 min at 72 °C; g. Hold at 4 °C). The gene product was electrophorized in 1% agarose gel. RT-qPCR was carried out using a 2× RealStar Green Fast Mixture with ROX II (GenStar) and run according to the program as described (a. 2 min at 95 °C; b. 15 s at 95 °C; c. 25 s at 55 °C; d. 30 s at 72 °C; e. Repeat steps b–d for 40 cycles).

The specific primers to selected genes were designed using Primer 5.0 software, and the sequences of the primers used are shown in Table 1 and Table 2.

### 2.4. Sodium Dodecyl Sulfate−Polyacrylamide Gel Electrophoresis (SDS−PAGE) and Western Blot Analysis

RSV-infected protoplasts treated with different concentrations of Ailanthone were extracted for total proteins by using a BCA protein Assay Kit (GenStar), which was applied to the Western blot immunoassay after SDS−PAGE. 

In brief, total proteins were separated by 12% SDS-PAGE and transferred to PVDF membranes. The membranes were blocked overnight at 4 °C and then inoculated with diluted anti-RSV CP-AP followed by FITC-conjugated goat anti-rabbit IgG. Signals were developed using the BCIP/NBT Alkaline Phosphatase Color Development kit (Biosharp, Beijing, China).

### 2.5. Curative, Protective, and Inactivating Activities of Ailanthone against the RSV Pathogenic Process in N. benthamiana

Ailanthone of a certain weight was dissolved in DMSO and then diluted with H_2_O to a final concentration as required in each test, and the amount of DMSO was no more than 0.5% in the final solution used in all of the tests. A solution containing the same amount of DMSO without Ailanthone was used as a control. 

*N. benthamiana* leaves were sprayed with a solution of 0.5 µM Ailanthone twice every 24 h. Selected leaves were inoculated with RSV extract 24 h after the final application of the test solution. Four days after virus inoculation, the leaf samples were collected and used for RNA isolation and RT-PCR analysis to investigate the protective effect of Ailanthone against RSV infection and replication in the *N. benthamiana* plant.

Selected leaves of *N. benthamiana* plants were inoculated with RSV extract. A solution of 0.5 µM Ailanthone was applied to the plants by spraying at 12 h and 36 h after virus inoculation, respectively. Four days after Ailanthone application, leaf samples were collected and used for RNA isolation and RT-PCR analysis to detect the curative effect of Ailanthone against RSV infection and replication in the *N. benthamiana* plant.

A mixture of RSV extract and Ailanthone solution was used for virus inoculation. Four days later, leaf samples were collected and used for RNA isolation and RT-PCR analysis to determine the inactivating effect of Ailanthone against RSV infection and replication in the *N. benthamiana* plant.

Leaf samples were always collected from three of the top fully expanded leaves of *N. benthamiana*. Each test was repeated three times.

### 2.6. Dose-Dependent Inhibitory Effects of Ailanthone on RSV Replication in N. benthamiana

The leaf disc method was employed to test the antiviral activities of Ailanthone against RSV replication in *N. benthamiana* at a series of gradient concentrations of 0.125, 0.25, 0.5, 0.75, and 1 µM. The whole fully expanded *N. benthamiana* leaf was mechanically inoculated with RSV extract. After 48 h, leaf disks (1 cm in diameter) were punched. Six leaf disks were randomly selected, floated on the Ailanthone solution in a Petri dish, and placed in a climate chamber at 25 °C. Leaf disks which were treated with a solution of 0.5% DMSO were used as controls. After 48 h, the leaf disks were used for RNA isolation and further prepared for RT-PCR analysis. A commercial antiviral agent, ningnanmycin, was used as a control agent, and tested using the same method at concentrations of 90 and 180 µM, respectively.

### 2.7. Dose-Dependent Inhibitory Effects of Ailanthone on RSV Replication in Rice Protoplasts

To develop an antiviral screening model, we must ensure that the tested agents exhibit no toxicity to rice protoplasts. Cell viability tests were conducted using a 3-(4, 5-dimethylthiazol-2-yl)-2, 5-diphenyl tetrazolium bromide (MTT) colorimetric assay to determine the toxic effects of Ailanthone on rice protoplasts. An MTT Cell Proliferation and Cytotoxicity Assay Kit ((Solarbio, Beijing, China)) was used in the test. The isolated rice protoplasts were incubated in WI buffer containing Ailanthone of a series of gradient concentrations of 0.125, 0.25, 0.5, 0.75, and 1 µM, respectively. The isolated rice protoplasts incubated in WI buffer without Ailanthone were used as controls. After incubation for 48 h, the rice protoplasts were tested for cell viability, and the result indicated that Ailanthone under the test concentrations showed no toxicity to rice protoplasts.

Thus, Ailanthone was dissolved with DMSO and then added to WI buffer to obtain solutions containing Ailanthone with a series of gradient concentrations of 0.125, 0.25, 0.5, 0.75, and 1 µM. The virus-infected protoplasts were resuspended in the Ailanthone-containing WI buffer and incubated in the dark at 28 °C for 48 h. A WI buffer with the addition of the same amount of DMSO as that of the test solutions was used as a control. Each treatment was repeated three times. The protoplasts were then used for RNA isolation and RT-PCR. Ningnanmycin was used as a control agent and tested using the same method at concentrations of 90 and 180 µM, respectively.

## 3. Results

### 3.1. Effect of Ailanthone on RSV Multiplication in N. benthamiana

#### 3.1.1. Dose-Dependent Inhibitory Activities of Ailanthone on RSV-CP Expression in *N. benthamiana*

**Figure 1:**
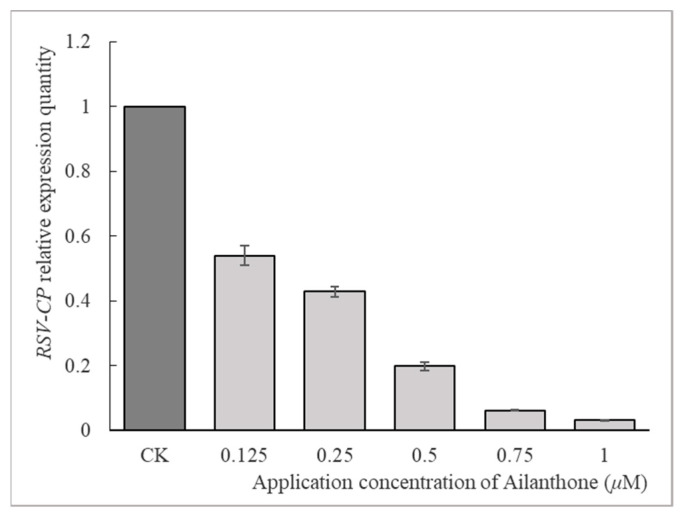
Dose-dependent inhibitory effect of Ailanthone on *RSV CP* expression in *N. benthamiana*. The data were from three independent experiments and the error bar represents ±SD.

The leaf disc method was employed to test the antiviral activities of Ailanthone against RSV at a series of gradient concentrations of 0.125, 0.25, 0.5, 0.75, and 1 µM. As shown in Figure 1, the relative expression quantities of *RSV CP*, which was representative of the RSV concentration in virus-infected *N. benthamiana*, were 0.54, 0.43, 0.20, 0.06, and 0.03, respectively. Ningnanmycin, a commercial antiviral agent, was applied and assayed using the same method as a control agent. The relative expression quantities of RSV *CP* are 0.42 and 0.17 when ningnanmycin was applied to virus-infected *N. benthamiana* at concentrations of 90 and 180 µM, respectively. The quantitative real-time polymerase chain reaction (RT-qPCR) results indicated that Ailanthone had a dose-dependent inhibitory effect against the *NSvc3* (*CP*) expression of RSV in *N. benthamiana*.

#### 3.1.2. Preventive, Inactivating, and Curative Effects against RSV Multiplication in *N. benthamiana*

**Figure 2:**
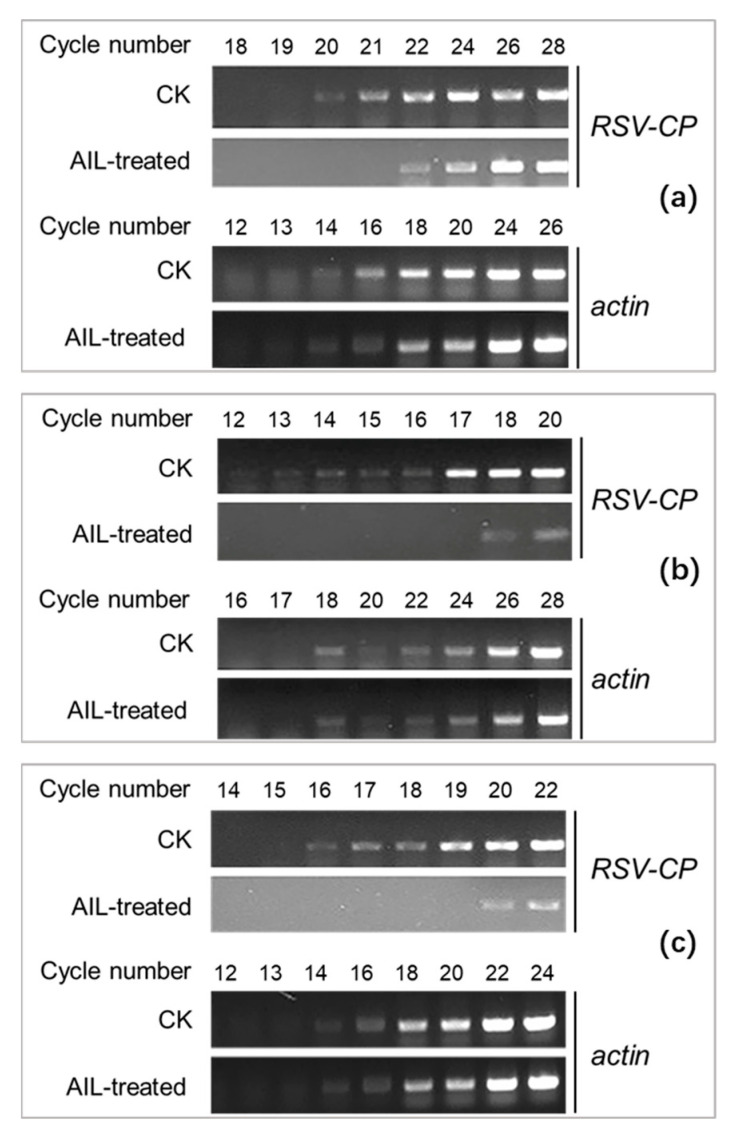
Semi-quantitative RT-PCR detection of *RSV-CP* gene expression in rice stripe virus-infected *N. benthamiana* treated with Ailanthone using different application methods. Ailanthone at a concentration of 0.5 µM was applied to *N. benthamiana* together with (**a**), pre- (**b**) or post-virus inoculation (**c**), and leaf samples were collected 4 d after the final treatment and used for RNA isolation and RT-PCR analysis.

A subsequent assay using the RSV-*N. benthamiana* system was designed to investigate whether Ailanthone could inhibit the virus pathogenic process at a specific stage, such as blocking the virus from infecting and entering the host plant, inactivating and weakening virus vitality, or restricting virus replication in plant cells. Ailanthone was applied to *N. benthamiana* together with pre- or post-virus inoculation, separately, to determine its inactivating, protective, or curative effect against RSV multiplication. Semi-quantitative RT-PCR detection of RSV-CP gene expression in *N. benthamiana*, as shown in Figure 2, indicated that 0.5 µM Ailanthone possessed a potent inhibitory effect against RSV in all three different application modes. The reference gene *actin* in both the control and treatment groups showed clearly visible bands starting from the same amplification cycle. The difference in *RSV CP*-specific amplification band abundance between the control and Ailanthone treatment groups indicated that *RSV-CP* gene expression was potently inhibited by Ailanthone at a concentration of 0.5 µM. Thus, the presented evidence showed that regardless of when it was applied (prior to or post virus infection), Ailanthone could always exhibit a potent interference or preventative effect against the pathogenic process of RSV, and thus reduce viral multiplication in RSV-infected *N. benthamiana*.

#### 3.1.3. Effects of Ailanthone on Coding Gene Expression of RSV in *N. benthamiana*

**Figure 3:**
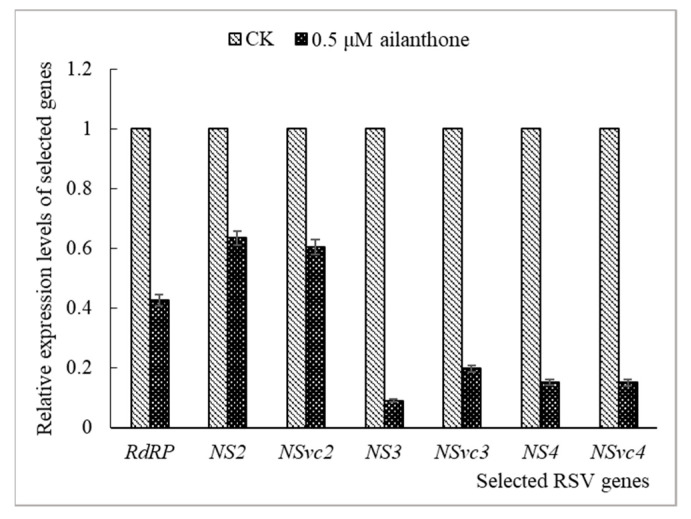
Effects of Ailanthone on RSV protein-coding gene expression in *N. benthamiana*. The data were from three independent experiments and the the error bar represents ±SD.

The RSV genome consists of four RNAs and encodes seven proteins, namely RNA-dependent RNA polymerase (RdRp), NS2, NSvc2 (glycoprotein), NS3 (gene-silencing suppressor), NSvc3 (coat protein, CP), NS4 (disease-specific protein, SP), and NSvc4 (movement protein, MP) [16,17]. Specific primers were designed as shown in detail in Table 2, and RT-qPCR was carried out to detect the influence of Ailanthone on RSV protein-coding gene expression in virus infected *N. benthamiana*. The expression of *RdRP*, *NS2*, *NSvc2*, *NS3*, *NS4*, and *NSvc4* in virus-infected *N. benthamiana* were all potently inhibited by 0.5 µM Ailanthone, and the relative expression quantities were 0.42, 0.63, 0.60, 0.09, 0.20, 0.16, and 0.15 (Figure 3), respectively. The results indicated that application of 0.5 µM Ailanthone could significantly inhibit the expression of seven protein-coding genes of RSV in *N. benthamiana* by different degrees.

### 3.2. Effect of Ailanthone on RSV Replication in Rice Protoplasts

#### 3.2.1. Dose-Dependent Inhibitory Activities of Ailanthone against RSV Multiplication in Rice Protoplasts

**Figure 4:**
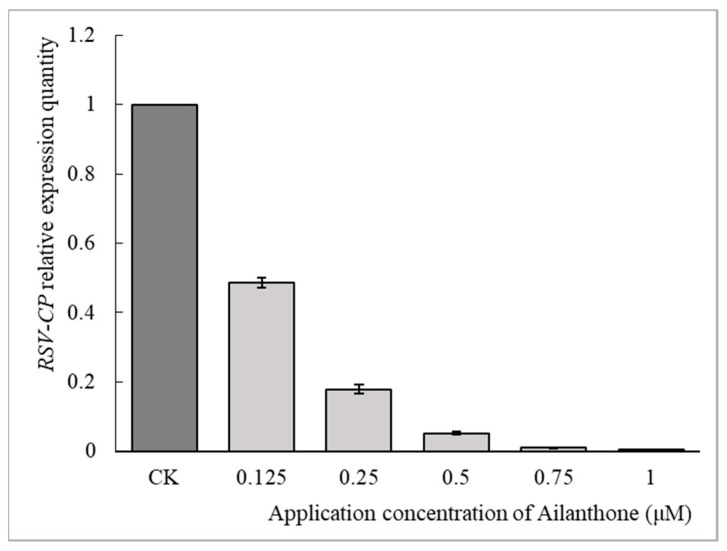
Dose-dependent inhibitory effects of Ailanthone on *RSV CP* expression in rice protoplasts. The data were from three independent experiments and the error bar represents ±SD.

Ailanthone of a series of gradient concentrations of 0.125, 0.25, 0.5, 0.75, and 1 µM was applied to RSV-infected rice protoplasts, and the RT-qPCR method was used to determine the relative expression of *RSV CP*. As shown in Figure 4, the relative expression quantities of *RSV CP* in rice protoplasts were 0.49, 0.18, 0.05, 0.01, and 0.003, respectively. The separate application of Ningnanmycin at concentrations of 90 and 180 µM also showed a potent inhibitory effect against *CP* expression, and the relative quantities are 0.28 and 0.07, respectively. The result revealed a dose-dependent inhibitory effect of Ailanthone against the virus multiplication of RSV in rice protoplasts.

**Figure 5:**
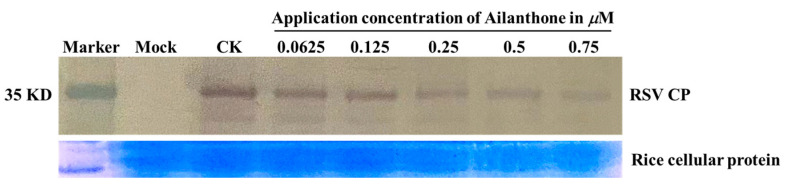
Western blotting of RSV CP in virus-infected rice protoplasts. Lane 1, protein marker; lane 2, rice protoplasts without RSV inoculation as a mock; lane 3, blot of RSV CP in rice protoplasts 48 h post-RSV inoculation as a control; lanes 4 to 8, blots of RSV CP in rice protoplasts 48 h post-RSV inoculation. The rice protoplasts were incubated with 0.0625, 0.125, 0.25, 0.5, and 0.75 µM of Ailanthone. The extracted rice protoplast protein was used as a loading control, which was separated by SDS-PAGE and stained with Coomassie Brilliant Blue as shown below the Western blots.

Western blot analysis was employed to further investigate the dose-dependent inhibition of Ailanthone on *RSV CP* expression in rice protoplasts. The target protein, RSV CP, can be detected at around 35 KD. The intensity difference of the distinct and specific bands of RSV CP, as shown in Figure 5, further confirmed the dose-dependent inhibitory effect of Ailanthone on *RSV CP* expression in rice protoplasts.

#### 3.2.2. Effects of Ailanthone on Coding Gene Expression of RSV in Rice Protoplasts

**Figure 6:**
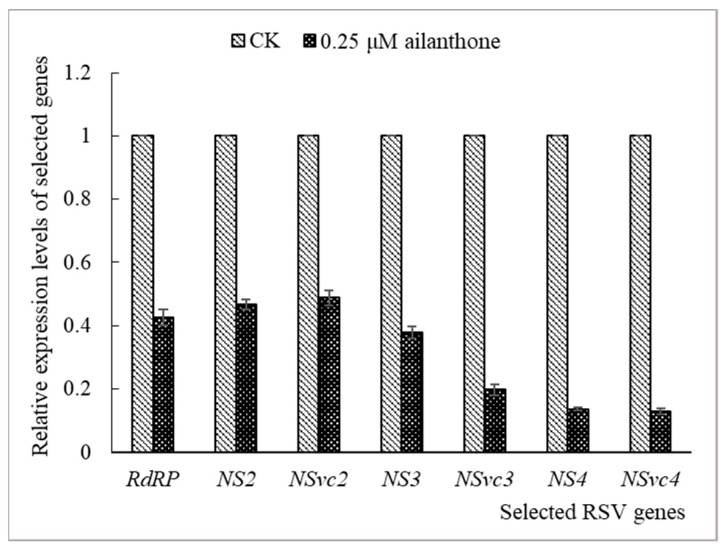
Effects of Ailanthone on RSV gene expression in rice protoplasts. The data were from three independent experiments and the error bar represents ±SD.

RT-qPCR was used to detect the influence of Ailanthone on the expression of seven protein-coding genes in RSV-infected rice protoplasts. The sequences of the specific primers used are shown in Table 2. Ailanthone at a concentration of 0.25 µM was applied to virus-infected rice protoplasts and incubated for 48 h, the relative expression quantities of *RdRP*, *NS2*, *NSvc2*, *NS3*, *NS4,* and *NSvc4* were 0.43, 0.47, 0.49, 0.38, 0.20, 0.14, and 0.13 (Figure 6), respectively.

## 4. Discussion

Quassinoids are a diverse class of highly oxygenated secondary metabolites, identified from natural plant materials mostly in the Simaroubaceae family, which are believed to be biosynthesized through the triterpenoid biogenetic pathway and originating from the oxidative degradation of tetracyclic tirucallane triterpene [18,19,20]. We initiated a phytochemistry study on the samara of *A. altissima* and reported the identification of more than 70 natural products of diverse structures, including quassinoids, coumarins, flavonoids, lignans, phenylpropanoids, phenylpropionamides, piperidine, and phenolic derivatives, among which were a series of novel compounds including eleven quassinoid glycosides, two phenylpropionamides, one piperidine, one terpenylated coumarin, and two phenolic derivatives [11,21,22,23]. Antiviral screening using the tobacco mosaic virus (TMV)-tobacco plant system revealed that quassinoids were the major active component from *A. altissima* secondary metabolites. Among the identified quassinoids, ailanthone and chapparinone showed the best antiviral activity against TMV. Further evidence revealed by Western blot analysis and green fluorescent protein (GFP)-tagged tobacco mosaic virus-based assays proved that Ailanthone could inhibit TMV *CP* expression and systematic spread of viruses in tobacco [11]. The crude MeOH extract of *A. altissima* stem barks has been proven to possess potent inhibitory effects against RSV in rice suspension cells [24]. As a highly active component and typical quassinoid from *A. altissima*, Ailanthone was selected and used to investigate its antiviral effects against the rice stripe virus in the present study. 

Rice stripe disease caused by the rice stripe virus is one of the most devastating plant diseases in rice, causing enormous losses in food production [25]. The re-emergence and outbreak of rice stripe disease in East Asia since 2000 have made RSV one of the most attractive focuses of research and attention. Extensive studies of RSV have resulted in substantial advances regarding fundamental aspects of the virus’s pathogenic process [26,27,28,29]. Screening of natural products derived from plant or microbial metabolites is still a promising strategy and approach for discovering lead compounds for the control of anti-plant virus diseases. The TMV-tobacco system is widely used for antiviral active natural product screening and has greatly driven the progression of the development of virus disease control agents [30,31,32,33,34]. 

Novel applicable methodology for the screening of antiviral agents is urgently needed to develop control methods for diverse, newly presented plant virus diseases. A new breakthrough in the development of screening methods for virus disease control agents is the report of the screening approach of anti-southern rice black-streaked dwarf virus agents based on *S7-1* gene expression in rice suspension cells [35]. The cap-snatching inhibitor screening approach was recently realized and developed based on the findings that cucumber mosaic virus (CMV) RNAs can serve as cap donors for the initiation of RSV transcription [36,37].

In the present study, an applicable and convenient leaf disc method was developed based on the systemic infection of RSV in *N. benthamiana*, partially drawing on the experience of the frequently used leaf disc method in the screening of anti-TMV agents. 

Ailanthone was proven to possess a dose-dependent influence upon RSV-CP expression in both virus-infected *N. benthamiana* and rice protoplasts (Figure 1 and Figure 4) as revealed by RT-qPCR analysis. When applied to virus-infected N. benthamiana at a concentration of 0.5 µM or to virus-infected rice protoplasts at a concentration of 0.25 µM, Ailanthone could inhibit the relative expression of RSV CP in host plant cells by more than 80% (Figure 1 and Figure 4). The dose-dependent inhibitory effect on RSV CP accumulation in RSV-infected rice protoplasts was further detected by Western blotting (Figure 5). Whether it was applied before or after virus inoculation, Ailanthone could exhibit significant inhibition on the RSV replication in host *N. benthamiana* (Figure 2). The above evidence obtained under laboratory conditions proved that Ailanthone deserves further study as an agent candidate or lead structure for the development of a control agent for plant virus diseases caused by RSV.

The negative/ambisense RNA genome of RSV consists of four single-stranded RNA segments [38]. The largest segment, RNA1, has a negative-sense polarity and encodes RNA-dependent RNA polymerase (RdRp). The RSV segments RNA2, RNA3, and RNA4 are ambisense and each encodes two open reading frames (ORFs) in opposite orientations on the viral RNA (vRNA) and viral complementary RNA (vcRNA) [1]. The NS3 protein of RSV, encoded by the viral strand of RNA3, is a viral suppressor of RNA silencing (VSR). NS3 plays a significant role in viral infection and NS3-transgenic plants manifest resistance to the virus [39]. Coat protein (NSvc3) of the rice stripe virus plays important roles in several biological processes, such as viral transcription and replication. It has been shown that overexpression of *RSV CP* in rice plants enhances resistance against virus infection [9]. During viral infection, the nonstructural protein NS4 of RSV formed cytoplasmic inclusions in various tissues of viruliferous SBPH and played a critical role in viral spread through insect vectors [40]. NSvc4 belongs to the virus movement protein “30 K” superfamily and is responsible for virus movement. It has the ability to localize to the plasmodesmata and is capable of complementing the cell-to-cell movement of movement-deficient potato virus X (PVX) [41]. 

Specific primers were designed and RT-qPCR was carried out to detect the influence of Ailanthone on RSV protein-coding gene expression in virus-infected *N. benthamiana* and rice protoplasts. When 0.5 µM Ailanthone was applied to virus-inoculated *N. benthamiana* or 0.25 µM Ailanthone was applied to virus-infected rice protoplasts, the expressions of seven RSV protein-coding genes were all significantly inhibited by different degrees (Figure 3 and Figure 6). Among them, the expressions of *NS3*, *NSvc3*, *NS4,* and *NSvc4* were affected relatively more than the other three genes in general, which indicates a relatively greater inhibitory effect of Ailanthone on the accumulation of RNA3 and RNA4 segments than that of RNA1 and RNA2 in virus-infected *N. benthamiana* and rice protoplasts. The results indicated a significant inhibitory effect of Ailanthone on the accumulation of RSV RNAs and encoding gene expression that were closely related to virus infection, replication, and spread.

## 5. Conclusions

Ailanthone, one of the typical C_20_ quassinoids isolated from *A. altissima*, was proven to possess a significant inhibitory effect against RSV multiplication in both the *N. benthamiana* plant and rice protoplasts by means of the detection of its influence on coding gene expression and the accumulation of RSV protein using RT-PCR and Western blot analysis. The significant and highly efficient inhibitory effect on RSV replication in the host plant indicated that Ailanthone deserves to be further studied as a candidate or a valuable lead structure for the development of an agent for the control of plant virus diseases caused by RSV.

## Figures and Tables

**Table 1:** Primers for *NSvc3* in RSV and reference genes used in semi-quantitative RT-PCR.

Gene	Forward Primer 5′−3′	Reverse Primer 5′−3′	Length (bp)
*NSvc3* (*CP*)	GCAGGCTATGATGCTGCAAC	TTGTCAGACCACGCTCCTTC	510
*Actin* (*N. benthamiana*)	ACTGATGAAGATACTCACAGA	TGGAATTGTATGTGGTTTCAT	236

**Table 2:** Primers for selected genes in RSV and reference genes used in quantitative real-time RT-PCR.

Gene	Forward Primer 5′−3′	Reverse Primer 5′−3′	Length (bp)
*RdRp*	CAGTGAAGTGGAGGCTGCT	AGCAAGTCTGTGGCTCTCAA	166
*NS2*	TGGGATGCTGTGAGGAGTTCA	GGATCAGTTTCAGATGCTCAGT	174
*NSvc2*	GAGAGGGATGGAGTGGACAT	TGAGGTCCCATTGAGGGATA	180
*NS3*	TTCACATCGTCTGTGGGTTC	TGGAAGGGTGCCTAGATGAATG	163
*NSvc3* (*CP*)	TGCAGAAGGCAATCAATGACAT	TGTCACCACCTTTGTCCTCAA	150
*NS4* (*SP*)	CCTGTTAGGAGGTGAAGATGATGA	GCTCTCAGCCTTAGCCATCTTG	180
*NSvc4* (*MP*)	TGAAGGCCCATAGGAAAGCA	TGCGGAGGGTAGTTATTCCAC	153
*18S rRNA* (Nipponbare)	ATGATAACTCGACGGATCGC	CTTGGATGTGGTAGCCGTTT	169
*Actin* (*N. benthamiana*)	TGTGCTCAGTGGTGGCTCTA	GGTGCTGAGAGAAGCCAAGATA	163

## Data Availability

All the data used in this study are already provided in the manuscript in the required section. There are no underlying data available.
